# HIV, Gender, Race, Sexual Orientation, and Sex Work: A Qualitative Study of Intersectional Stigma Experienced by HIV-Positive Women in Ontario, Canada

**DOI:** 10.1371/journal.pmed.1001124

**Published:** 2011-11-22

**Authors:** Carmen H. Logie, LLana James, Wangari Tharao, Mona R. Loutfy

**Affiliations:** 1 Women's College Research Institute, Women's College Hospital, University of Toronto, Toronto, Ontario, Canada; 2 Women's Health in Women's Hands Community Health Centre, Toronto, Ontario, Canada; Medical Research Council, South Africa

## Abstract

**Background:**

HIV infection rates are increasing among marginalized women in Ontario, Canada. HIV-related stigma, a principal factor contributing to the global HIV epidemic, interacts with structural inequities such as racism, sexism, and homophobia. The study objective was to explore experiences of stigma and coping strategies among HIV-positive women in Ontario, Canada.

**Methods and Findings:**

We conducted a community-based qualitative investigation using focus groups to understand experiences of stigma and discrimination and coping methods among HIV-positive women from marginalized communities. We conducted 15 focus groups with HIV-positive women in five cities across Ontario, Canada. Data were analyzed using thematic analysis to enhance understanding of the lived experiences of diverse HIV-positive women. Focus group participants (*n* = 104; mean age = 38 years; 69% ethnic minority; 23% lesbian/bisexual; 22% transgender) described stigma/discrimination and coping across micro (intra/interpersonal), meso (social/community), and macro (organizational/political) realms. Participants across focus groups attributed experiences of stigma and discrimination to: HIV-related stigma, sexism and gender discrimination, racism, homophobia and transphobia, and involvement in sex work. Coping strategies included resilience (micro), social networks and support groups (meso), and challenging stigma (macro).

**Conclusions:**

HIV-positive women described interdependent and mutually constitutive relationships between marginalized social identities and inequities such as HIV-related stigma, sexism, racism, and homo/transphobia. These overlapping, multilevel forms of stigma and discrimination are representative of an intersectional model of stigma and discrimination. The present findings also suggest that micro, meso, and macro level factors simultaneously present barriers to health and well being—as well as opportunities for coping—in HIV-positive women's lives. Understanding the deleterious effects of stigma and discrimination on HIV risk, mental health, and access to care among HIV-positive women can inform health care provision, stigma reduction interventions, and public health policy.

*Please see later in the article for the Editors' Summary*

## Abstract 2

Mona Loutfy and colleagues used focus groups to examine experiences of stigma and coping strategies among HIV-positive women in Ontario, Canada.

## Editors' Summary

**Background:**

HIV-related stigma and discrimination—prejudice, negative attitudes, abuse, and maltreatment directed at people living with HIV—is a major factor contributing to the global HIV epidemic. HIV-related stigma, which devalues and stereotypes people living with HIV, increases vulnerability to HIV infection by reducing access to HIV prevention, testing, treatment, and support. At the personal (micro) level, HIV-related stigma can make it hard for people to take tests to determine their HIV status or to tell other people that they are HIV positive. At the social/community (meso) level, it can mean that HIV-positive people are ostracized from their communities. At the organizational/political (macro) level, it can mean that health-care workers treat HIV-positive people differently and that governments are deterred from taking fast, effective action against the HIV epidemic. In addition, HIV-related stigma is negatively associated with well-being among people living with HIV. Thus, among HIV-positive people, those who have experienced HIV-related stigma have higher levels of mental and physical illness.

**Why Was This Study Done?:**

Racism (oppression and inequity founded on ethno-racial differences), sexism and gender discrimination (oppression and inequity based on gender bias in attitudes), and homophobia and transphobia (discrimination, fear, hostility, and violence towards nonheterosexual and transgender people, respectively) can also affect access to HIV services. However, little is known about how these different forms of stigma and discrimination interact (intersect). A better understanding of the effect of intersecting stigmas on people living with HIV could help in the development of stigma reduction interventions and HIV prevention, treatment and care programs, and could help to control global HIV infection rates. In this qualitative study (an analysis of people's attitudes and experiences rather than numerical data), the researchers investigate the intersection of HIV-related stigma, racism, sexism and gender discrimination, homophobia and transphobia among marginalized HIV-positive women in Ontario, Canada. As elsewhere in the world, HIV infection rates are increasing among women in Canada. Nearly 25% of people living with HIV in Canada are women and about a quarter of all new infections are in women. Moreover, there is a disproportionately high infection rate among marginalized women in Canada such as sex workers and lesbian, bisexual, and queer women.

**What Did the Researchers Do and Find?:**

The researchers held 15 focus groups with 104 marginalized HIV-positive women who were recruited by word-of-mouth and through flyers circulated in community agencies serving women of diverse ethno-cultural origins. Each focus group explored topics that included challenges in daily life, medical issues and needs, and issues that were silenced within the participants' communities. The researchers analyzed the data from these focus groups using thematic analysis, an approach that identifies, analyzes, and reports themes in qualitative data. They found that women living with HIV in Ontario experienced multiple types of stigma at different levels. So, for example, women experienced HIV-related stigma at the micro (“If you're HIV-positive, you feel shameful”), meso (“The thing I hate most for people that test positive for HIV is that society ostracizes them”), and macro (“A lot of women are not getting employed because they have to disclose their status”) levels. The women also attributed their experiences of stigma and discrimination to sexism and gender discrimination, racism, homophobia and transphobia, and involvement in sex work at all three levels and described coping strategies at the micro (resilience; “I always live with hope”), meso (participation in social networks), and macro (challenging stigma) levels.

**What Do These Findings Mean?:**

These findings indicate that marginalized HIV-positive women living in Ontario experience overlapping forms of stigma and discrimination and that these forms of stigma operate over micro, meso, and macro levels, as do the coping strategies adopted by the women. Together, these results support an intersectional model of stigma and discrimination that should help to inform discussions about the complexity of stigma and coping strategies. However, because only a small sample of nonrandomly selected women was involved in this study, these findings need to be confirmed in other groups of HIV-positive women. If confirmed, the complex system of interplay of different forms of stigma revealed here should help to inform health-care provision, stigma reduction interventions, and public-health policy, and could, ultimately, help to bring the global HIV epidemic under control.

**Additional Information:**

Please access these Web sites via the online version of this summary at http://dx.doi.org/10.1371/journal.pmed.1001124.

- Information is available from the US National Institute of Allergy and Infectious Diseases on HIV infection and AIDS
- NAM/aidsmap basic information about HIV/AIDS, and summaries of recent research findings on HIV care and treatment; its publication HIV and stigma deals with HIV-related stigma in the UK
- Information is available from Avert, an international AIDS charity on many aspects of HIV/AIDS, including information on women, HIV, and AIDS, on HIV and AIDS stigma and discrimination, and on HIV/AIDS statistics for Canada (in English and Spanish)
- The People Living with Stigma Index to address stigma relating to HIV and advocate on key barriers and issues perpetuating stigma; it has recently published Piecing it together for women and girls, the gender dimensions of HIV-related stigma; its website will soon include a selection of individual stories about HIV-related stigma
- Patient stories about living with HIV/AIDS are available through Avert and through the charity website Healthtalkonline

## Introduction

Mirroring global HIV statistics, HIV infection rates are increasing among women in Canada [1],[2]. As of 2008, women constitute 22% of people living with HIV (PLHIV) within Canada and 26% of new HIV infections—an 11% increase from the previous year [1]. Of particular concern are the disproportionate infection rates among marginalized women. For example, there is a 7-fold over-representation of new HIV infections in the Black female population in Canada in comparison with the general population. This substantial health inequity underscores the necessity of understanding the factors driving the disproportionate impact of HIV among marginalized women in Canada.

Stigma and discrimination are principal factors contributing to the HIV epidemic. For example, HIV-related stigma increases vulnerability to HIV infection by reducing access to HIV prevention and testing and presenting barriers to treatment, care, and support for PLHIV [3]–[8]. Stigma refers to processes of devaluing, labeling, and stereotyping that are manifested in the loss of status, unfair and unjust treatment, and social isolation of individuals or groups [9]–[12].

Stigma may be based on multiple aspects of one's actual or perceived identity or group membership, such as HIV-positive serostatus, race and ethnicity, and sexual orientation. Key issues identified in previous research with populations at elevated risk for HIV infection in Canada include: HIV-related stigma, racism, sexism and gender discrimination, homophobia, and transphobia. HIV-related stigma refers to the devaluing of people who are HIV-positive or associated with HIV and AIDS and may result in discrimination based on actual or perceived HIV-positive serostatus [12]. Numerous studies across the globe have documented negative associations between HIV-related stigma and well being of PLHIV. For example, a meta-analysis of 24 studies conducted with PLHIV in North America documented correlations between higher rates of HIV-related stigma and higher levels of deleterious mental and physical health outcomes [13].

Racism, another form of stigma and discrimination, refers to systems of oppression and inequity founded on ethno-racial differences, including sociocultural beliefs, attitudes, exclusion, harassment, and institutional policies and practices [14],[15]. Jones (2000) described three forms of racism: institutionalized (unequal access to material conditions and opportunities), personally mediated (intentional and unintentional prejudice and discrimination), and internalized (a stigmatized person's acceptance of negative messages about oneself and one's community) [15],[16]. Racism has been associated with deleterious physical and mental health outcomes and reduced well being in numerous studies [17]–[20]. A systematic review of 138 population-based studies examining self-reported racism and health highlighted the associations between higher levels of racism and increased mental health (e.g., depression) and physical health (e.g., increased blood pressure) issues [21]. Higher HIV infection rates among African Americans in the US in comparison with the general population have been attributed to institutionalized racism (e.g., reduced access to sexual health care clinics, disproportionate incarceration rates among African Americans) [22].

Sexism and gender discrimination may in part account for physical and mental health inequities between women and men [23]–[27]. Sexism and gender discrimination refer to systems of oppression and inequity based on gender bias in attitudes, treatment, sociocultural values, harassment, violence, and institutional policies and practices [14],[28]. Self-reported sexism has been associated with deleterious mental health outcomes among women, such as increased depression, anxiety, and eating disorders [23]–[27]. Gender inequity may enhance HIV infection risks by reducing women's ability to negotiate safer sex with male partners [29].

Homophobia and transphobia refer to discrimination, fear, hostility, and violence towards nonheterosexual and transgender people, respectively [10]. The stress resulting from stigma and discrimination towards lesbian, gay, bisexual, transgender, and queer people has been attributed to increased mental health diagnoses among these populations [30]–[32]. Homophobia and transphobia may reduce access to HIV prevention services and contribute to sexual violence—both increase HIV infection risk among sexual minorities and transgender people [33].

**Figure 1:**
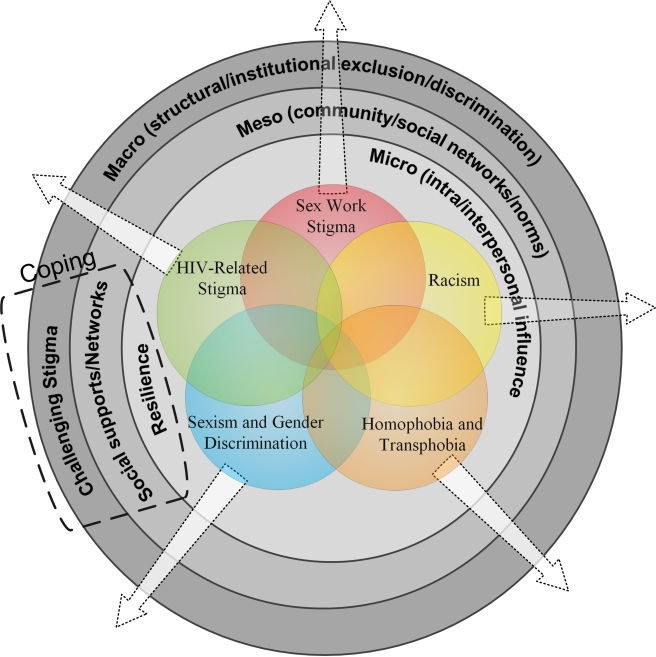
Conceptual model of intersectional stigma and coping among women living with HIV (*n* = 104) in Ontario, Canada. Focus groups (*n* = 15) with diverse women living with HIV (*n* = 104) in five cities across Ontario, Canada, revealed experiences of multilevel (micro, meso, macro), intersecting forms of stigma (HIV-related stigma, racism, homophobia and transphobia, sexism and gender discrimination, sex work stigma). Participants also highlighted multilevel coping strategies, including resilience (micro), social networks (meso), and challenging stigma (macro).

HIV-related stigma analyses are not only complicated by the intersection with other forms of stigma, but also by the multiple levels of stigma. Stigmatizing processes operate on multiple levels: micro (e.g., individual attitudes and beliefs), meso (e.g., community and social norms/networks), and macro (e.g., structural factors including organizational and political power; laws and policies; health and social service systems) [7],[9],[21],[34]–[36] (Figure 1). At the micro-level, individual levels of influence include intrapersonal (individual characteristics such as knowledge, beliefs, and attitudes) and interpersonal (relations with family and friends) dimensions [35].

This substantial evidence highlights the alarming health impacts of HIV-related stigma, racism, sexism, homophobia, and transphobia, yet considerably less attention has been given to understanding the intersection of these forms of stigma. A key facet missing from most HIV-related stigma research is the association between HIV-related stigma and other forms of stigma. A recent HIV-related stigma systematic review [7] and meta-analysis [13] highlighted that most research has examined these forms of stigma and discrimination separately. For example, in a meta-analysis of 24 studies examining health and demographic correlates of HIV-related stigma among PLHIV in North America, only two studies examined race/ethnicity and three examined gender [13]. Mahajan and colleague's (2008) systematic review of HIV-related stigma recommended that future research develop a comprehensive conceptual model for HIV-related stigma that includes overlapping stigma associated with race, gender, and sexual orientation. The present study endeavors to develop such a model utilizing the concept of intersectionality [7].

Intersectionality refers to the interdependent and mutually constitutive relationship between social identities and structural inequities [37]–[39]. This perspective views the intersection of identities as synergistic, producing different and distinct experiences of oppression and opportunity [38],[40]. Examining the intersection of stigmas is particularly relevant in the context of HIV-related stigma that exacerbates pre-existing structural inequities based on race, class, gender, and sexual orientation [34]. Some researchers have employed an intersectional theoretical approach to explore overlapping stigmas experienced by HIV-positive women in the US and UK [41]–[43]: study findings suggest that racism and sexism converge with HIV-related stigma to negatively impact health outcomes among PLHIV. Intersectionality warrants further integration in HIV-related stigma research and conceptualizations [7],[44]. Qualitative research findings indicate that HIV prevention barriers among Black women in Canada include HIV-related stigma, sexism, racism, and homophobia [3]–[6]—highlighting the salience of an intersectional approach.

An enhanced understanding of the experiences of HIV-positive women who face intersecting stigmas can inform stigma reduction interventions, health promotion initiatives, and enrich understanding of stigmatizing processes. This study is guided by an intersectional analysis that moves beyond a single-axis framework of discrimination to look at multiple categories of difference and the experiences of people at the intersections of these differences [38],[45],[46]. Specifically, this study aimed to explore the intersection of HIV-related stigma, racism, sexism and gender discrimination, homophobia, and transphobia (Figure 1).

The objectives of this study were to enhance understanding of stigma and coping strategies among women living with HIV in Ontario, Canada. Ontario was selected as a study site as it holds the highest proportion (44.4%) of Canada's 65,000 PLHIV [1]. We explored the following two research questions: (1) How do women living with HIV in Ontario experience stigma and discrimination? (2) What strategies do women living with HIV use to cope with stigma and discrimination?

## Methods

### The Study

A community-based qualitative investigation was conducted in partnership between an urban community health center serving women of colour and a research institute focused on women's health. A community advisory board (CAB) composed of members (*n* = 11) from agencies providing health/social services to HIV-positive women was developed and met five times over 2 y to provide consultation on research design, implementation, and interpretation of results. The project team included peer research assistants (PRA) who were HIV-positive women who represented the diversity of women included in focus groups. PRA attended a 2-d training on research methods and assisted with: pilot testing the focus group guide, participant recruitment, cofacilitation of focus groups, and member-checking results. Training and involving PRA at multiple stages of the research process promotes capacity-building and the Greater Involvement of People Living with HIV principle [47].

### Data Collection

Fifteen focus groups (12 English, two French, one Spanish) were held with diverse HIV-positive women in five cities across Ontario (see Table 1 below). Focus groups help to promote community involvement, in-depth probing, and are particularly appropriate for understanding how people interpret experiences [48]–[50]. It was also the most feasible and cost-efficient approach to include many populations and locations in this study. Inclusion criteria for focus group participants included adults 18 y of age and older who self-identified as women living with HIV.

**Table 1:** Overview of focus groups (*n* = 15).

Focus Group Population	Location	*n*
Transgender women	Toronto	16
African Caribbean women	Toronto	11
Women (industrial, medium-sized city)	Hamilton	9
African Caribbean women	Ottawa	9
Women with experiences of incarceration	Toronto	8
IDU women	Toronto	7
Francophone	Toronto	7
Sex workers	Toronto	7
LBQ women	Toronto	7
HIV-positive women service providers	Toronto	6
Young women (18–24 y)	Toronto	5
Asian and South Asian	Toronto	5
Women (Northern Ontario, small-city)	Thunder Bay	4
Women (Northern Ontario, town)	Sudbury	2
Latina	Toronto	1

Purposive and convenience sampling were used given the nature of the study population and no sampling frame of diverse HIV-positive women in Ontario [50],[51]. Specifically, in this study participants were recruited via word of mouth and flyers in community agencies, including AIDS service organizations (ASO), lesbian/bisexual/queer/transgender [LBQT] community centers, and ethno-specific social/health service agencies serving women of diverse ethno-cultural origins. PRA also shared information about the focus group to their social and community networks. Participants registered for the focus groups via phone or email; staff at community agencies also registered women interested in participating.

Focus groups were conducted at various venues that provided services to the focus group population; for example, ASO, community health centers, a harm reduction centre, and ethno-cultural agencies. Focus groups were 120 min in duration and cofacilitated by a PRA and a research coordinator (CHL) who was a doctoral candidate. Prior to the focus group written informed consent was obtained from participants and a brief information sheet was distributed to collect sociodemographic information. No identifying information was collected from participants. Information about local resources for HIV-positive women was disseminated at the end of each focus group. Participants received a CDN$50.00 honorarium for time and travel. The Research Ethics Board at Women's College Hospital in Toronto approved the study.

Challenges emerged in recruitment of focus group participants: the aim was to recruit six to ten participants per focus group. However, as displayed in Table 1 below, some groups were oversubscribed (i.e., transgender women with 16 participants) while others were undersubscribed. For example, despite numerous attempts and rescheduling, only one woman participated in the Latina focus group and five participated in the Asian/South Asian group. This situation could be reflective of the lack of services geared for Latina, Asian, and South Asian HIV-positive women—the culturally specific ASOs predominately serve men. In contrast, the PRA advertised the transgender focus group at a transgender drop-in centre in close proximity to the focus group location (Table 1).

We used a semistructured focus group interview guide with open-ended questions to explore strengths and challenges experienced by women living with HIV in Ontario. Each focus group explored the following topics: research priorities (e.g., important issues in the lives of HIV-positive women); challenges and strengths in daily life; medical issues and needs; community and academic partnerships (e.g., relationships between participants and university researchers); and issues that were silenced in one's community. The current analysis focuses on the responses regarding stigma, discrimination, and coping strategies.

### Data Analysis

Focus groups were digitally recorded, transcribed verbatim, and entered into NVivo 8 qualitative analysis software. French and Spanish focus groups were transcribed in French/Spanish and translated into English for data analysis. Data were analyzed using thematic analysis, a method used to identify, analyze, and report themes in data [52]. Thematic analysis can be applied across diverse theoretical approaches and incorporates both inductive and deductive analyses [52]. In the present study, inductive analyses were used to identify new themes that evolved in the data, and deductive approaches were used to explore themes identified by the intersectional theoretical approach guiding the study (e.g., HIV-related stigma, sexism, racism, homophobia, transphobia).

Two investigators (CHL, LJ) conducted thematic analyses: reading the transcripts several times and recording preliminary ideas; producing initial codes by highlighting relevant data (e.g., instances of stigma); generating themes by collating codes across the data set (e.g., stigma associated with HIV); reviewing themes to develop a thematic map (e.g., intersection of multiple types of stigma); refining themes (e.g., highlighting the multiple levels of stigma); and generating the final report by selecting examples of themes and relating findings to the literature [52].

These investigators met to discuss each stage of analysis and were in 85% agreement with the final coding framework. Discrepancies between coding were resolved in team meetings among the four investigators (CHL, LJ, WT, MRL). No major new themes emerged after coding the data from focus group discussions, indicating that saturation had been attained. Initial data analysis and findings were presented at meetings with the PRA and CAB as a form of member checking [48],[53]. The PRA and CAB members were in agreement with the findings and analysis therefore no back and forth was carried out.

## Results

Sociodemographic characteristics of focus group participants (*n* = 104) are reported below in Table 2. The mean participant age was 38 y (standard deviation 9.1), over half of participants (62.4%) reported an annual income below CDN$19,999, and the majority of participants (61.5%) were born outside of North America (Table 2).

**Table 2:** Sociodemographic characteristics of focus group participants (*n* = 104).

Characteristic	Value or Percent	*n*
**Age, y**		
Range	20–57	96
Mean	38.3 (SD 9.1)	96
**Annual income (** ***n*** ** = 93)** a		
CDN$0–CDN$19,999.00	62.4	58
CDN$20,000–CDN$39,999.00	31.2	29
>CDN$40,000.00	6.6	6
**Highest level of education (** ***n*** ** = 96)** a		
Some high school or less	29.2	28
High school	22.9	22
Some college/university	21.9	21
Completed college/university	21.9	21
Graduate degree	4.2	4
**Sexual orientation/gender identity (** ***n*** ** = 96)** a		
Heterosexual	77.1	74
Bisexual	15.6	15
Queer	4.2	4
Lesbian	3.1	3
Transgender	21.9	21
**Region of birth (** ***n*** ** = 96)** a		
North America	38.5	37
Africa	36.5	35
Caribbean	9.4	9
Mexico, Central and South America	7.3	7
Europe	5.2	5
Asia/South Asia	3.1	3
**Ethnicity (** ***n*** ** = 103)** a **(participants could list more than 1)**		
African	31.6	37
European	18.0	21
Aboriginal	16.5	17
“Canadian”	14.6	15
Latina	3.4	4
Asian	2.6	3
South Asian	2.6	3

a The *n* is lower due to missing responses.

This study aimed to explore experiences of stigma/discrimination and coping among diverse HIV-positive women. On the basis of previous literature, it was hypothesized that women would experience stigma based on HIV-positive serostatus, gender, race/ethnicity, gender identity, and sexual orientation. Inductive coding resulted in identifying an additional source of stigma (involvement in sex work) and multiple levels (micro, meso, macro) (Figure 1) in which coping and stigma processes operated.

### Experiences of Stigma Experienced by Women Living with HIV in Ontario

Findings highlight the intersection of stigma across micro, meso, and macro levels: HIV-related stigma; sexism and gender discrimination; racism; homophobia and transphobia; and sex work stigma. Table 3 highlights the focus groups that discussed each theme.

**Table 3:** Overview of focus group participants' (*n* = 104) descriptions of stigma and discrimination.

Theme	Focus Groups Reporting Theme	Illustrative Quotations
HIV-related stigma	1, 2, 3, 4, 5, 6, 7, 8, 9, 10, 11, 12, 13, 14, 15	*“If you're HIV-positive you feel shameful”* (transgender participant);*“The thing I hate the most for people that test positive for HIV is that society ostracizes them”* (IDU participant); *“A lot of women are not getting employed because they have to disclose their status”* (South Asian participant)
Sexism and gender discrimination	1, 2, 3, 4, 5, 8, 9, 10, 11, 13, 14, 15	*“Women with HIV often end up in abusive relationships, suffer from violence”* (African Caribbean participant, Toronto); *“It's more acceptable for a man to have HIV than a woman. Women, they're looked at like they're dirty”* (sex worker participant)*;“Sometimes a woman cannot get out of the relationship. If the husband has papers [immigration] but not the woman, when it comes to violence you cannot leave him”* (African Caribbean participant, Ottawa)
Racism	1, 2, 5, 8, 13, 15	*“I can go on for days with the racism. Everything just builds up”* (African Caribbean participant, Toronto); *“I need you to listen me and to help me. But you're thinking ‘you come from Africa you don't understand’”* (young woman participant); *“As an Aboriginal when you are accessing ASOs there is big phobia”* (Aboriginal transgender participant)
Homophobia and transphobia	6, 8, 11, 12, 13	*“The stigma is double: it's stigma because you're HIV, stigma that you're not straight, so the pressure is just too much to handle”* (LBQ participant); *“Automatically if you're HIV-positive, you're gay”* (transgender participant); *“The HIV prevention course only focuses on heterosexual people. So you are just left out”* (LBQ participant)
Sex work stigma	11, 13	*“I've been positive for almost ten years. And I don't even tell half the people I know. As soon as I tell them they distance themselves”* (sex worker); *“Most people think that prostitutes are disease-carrying people”* (sex worker)

Focus Group Key: 1, African Caribbean (Ottawa); 2, African Caribbean (Toronto); 3, Asian and South Asian; 4, formerly incarcerated; 5, Francophone; 6, IDU; 7, Latina; 8, LBQ; 9, Northern Ontario medium-sized city; 10, Northern Ontario small-sized city; 11, sex work; 12, South Western Ontario medium-sized city; 13, transgender; 14, urban; 15, young women.

### HIV-Related Stigma: “It's Such a Taboo Topic”

Participants discussed experiences of multiple types of stigma (felt-normative, internalized, enacted stigma, symbolic) associated with being HIV-positive that were manifested across micro, meso, and macro levels of influence.

Micro level forms of stigma were described in both intrapersonal and interpersonal domains. Intrapersonal levels of stigma included intense shame and internalized stigma; internalized stigma refers to a stigmatized individual's acceptance of negative beliefs, views, and feelings towards the stigmatized group and oneself [21]. As a transgender participant described: “Accepting yourself with HIV is hard. It's really hard. It's hard to live with it. It's hard to have a normal life.” The severe consequences of intrapersonal stress caused by internalized stigma are evidenced in another narrative: “the first thing you think of is how yucky you are, and then some people think suicide. I know a few people that have killed themselves” (Northern Ontario, small city).

Participants also discussed hiding their HIV-positive serostatus from friends and family to manage felt-normative stigma in interpersonal domains. Felt-normative stigma refers to awareness of negative societal attitudes and fear of discrimination [10],[21],[32]. As a young woman stated: “I've had HIV for five years now. My parents don't know and I'm scared to tell them, because my mom will have a heart attack”.

Participants described that when they did disclose their HIV-positive serostatus they often experienced enacted stigma in interpersonal spheres. Enacted stigma encompasses overt acts of discrimination, such as social exclusion and violence [10],[54]. A young woman explained: “You think they're close friends, you decide to open to them and say, ‘I have HIV’. But then they don't want anything to do with you anymore. They stop calling, they stop coming over.” Thus participants found it difficult to accept their own HIV-positive serostatus and often hid their serostatus to avoid upsetting family and friends. This nondisclosure caused intrapersonal stress: “inside of me I'm dying of sadness, having to hide this” (African Caribbean).

HIV-related stigma was described as embedded in community and social norms (meso level). Symbolic stigma refers to othering, blaming and shaming of a marginalized group (e.g., PLHIV) and people associated with this stigmatized group (e.g., sex workers) [35],[36]. For example, symbolic HIV-related stigma frames HIV as a “dirty” and “immoral” disease. A sex worker explained: “cancer doesn't have as much as stigma as AIDS or HIV. Because it's sexually transmitted people look at it like you're dirty, you're not clean, bringing it on yourself.” This account suggests that the sexually transmitted nature of HIV differentiates it from other diseases and results in the attribution of blame towards PLHIV. Felt-normative HIV-related stigma constructs PLHIV as dangerous: “you're labeled as the pest of the society because you're infected” (Latina). This stigma may contribute to the silence regarding HIV: “There is a fear, denial and a total silence in the community about it” (South Asian).

This fear and silence regarding HIV present barriers to disclosing one's HIV-positive serostatus: “It's such a taboo topic, so it's really hard to have an open conversation about HIV. Because you don't know how that person is going to perceive you” (sex worker). These fears of mistreatment may be justified as participants described widespread enacted societal stigma. An African Caribbean participant explained: “discrimination is everywhere once they know you have HIV.” Other participants described being “ostracized” (injection drug using [IDU]) and “treated like garbage” (formerly incarcerated).

Enacted HIV-related stigma was also experienced in social services and health care domains (macro level). This stigma was at times subtle, and other times overt. A sex worker described: “once my ODSP [disability] worker found out that I was HIV-positive, she was just different. Her attitude changed.” A Francophone African woman expressed frustration with health care treatment: “I was in crisis—I had to go see a doctor. When she came in she had three pairs of gloves and yet it wasn't even a problem related to HIV! She kept her distance and could barely touch me. When she finally came closer I said: ‘If you do not remove your gloves you do not touch me!’”

### Sexism and Gender Discrimination: “He Was Going to Take My Son Away”

Sexism and gender discrimination were highlighted in relationships and societal attitudes and participants highlighted a dearth of women-specific HIV services. Participant stories suggest that internalized HIV-related stigma may render it difficult to leave abusive interpersonal relationships (micro level). A young woman described: “when you have HIV, you want to be loved unconditionally. Fear of leaving the relationship, that you wouldn't find another person to love you, to treat you the way that he was treating you before, you take abuse.” Internalized stigma may present a barrier to leaving unhealthy relationships: “a lot of the times people blame themselves for being abused” (sex worker).

Other participant accounts indicate that their HIV-positive status was exploited in inequitable relationships with male partners. A participant (Northern Ontario, small city) recounted: “I did not tell my ex [I was HIV-positive]; I knew he was going to take my son away. Somebody wrote him a letter and said ‘keep your son away from his mother, she's HIV positive.’ My son was told by his daddy he's going to catch AIDS.” Community and social (meso level) norms may silence discussion regarding violence against HIV-positive women. An African Caribbean participant explained: “Women with HIV often end up in abusive relationships, suffer from violence. People don't want to talk about that. There's no program specific to women with HIV in violent relationships.”

Sexism and gender discrimination were highlighted in employment systems (macro level). A sex worker explained: “I lost my job because my manager stalked me. You know, just being a woman in general and then working in the sex trade—you can't demand respect in the health care system or in the employment system.” Participant narratives highlighted a lack of women-specific programs and services at ASO (macro level). An African Caribbean participant described that this exclusion may be due to perceptions of HIV as a “gay man's” disease: “most of these ASO do not cater specifically for women. I think that comes from the fact that long back HIV and AIDS was well known to be a white gay man's disease.”

### Racism: “You're Thinking ‘You Come from Africa, You Don't Understand’”

Racism was highlighted in daily life, research, HIV services, and health care. An Aboriginal participant articulated: “We are suppressed as natives in our nation” (Northern Ontario, town); this invokes the perception that racism is a phenomenon of daily life. Such daily experiences of racism often resulted in intrapersonal stress (micro level): “I can go on for days with the racism. Everything just builds up” (African Caribbean).

Participants' narratives highlighted community and social norms (meso level) that exclude and silence the voices of people of color. A Francophone African woman described that with regard to HIV services, “The African community, for example, we are not informed about anything. Nothing at all.” These limited opportunities for Africans to participate in HIV programs suggest institutionalized racism in HIV services. Another participant expressed: “women of color are silent about their needs and what they want exactly from an ASO because we think it can't be changed. They're afraid if they say anything, it will be taken from them” (African Caribbean). This narrative reflects the experiences of women of color who may feel disempowered in HIV service delivery because of the intersection of race/ethnicity and gender.

Enacted stigma was discussed in research and health care (macro level). A participant articulated: “research is all about the White folks and what the White folks want to get from the Black people” (African Caribbean), highlighting the perception that white researchers exploit black bodies. Institutionalized racism was also noted in health care and pregnancy planning. For example, a participant described differential treatment at a fertility clinic: “Because you are black, if you go to the clinic and you say you wanna have a baby, they say, ‘Why do you wanna have a baby when you know your situation?’ Well they don't say that to the white people” (African Caribbean). This account suggests that the interaction of race/ethnicity and HIV serostatus may result in less support in pregnancy planning for black HIV-positive women in comparison with white HIV-positive women.

### Homophobia and Transphobia: “They See You Being Queer as Being Demonic”

Participants recounted experiences of homophobia and transphobia within familial, community, and health care institutions. In the interpersonal sphere (micro level), participants described experiences of familial violence: “in my community they know that I'm bisexual. I got my HIV through my brothers, they gang raped me” (lesbian, bisexual, and queer [LBQ]). On an intrapersonal level, participants' stories highlighted the convergence of HIV-related stigma and homophobia was difficult to cope with. One woman reflected: “I think the stigma is double—it's stigma because you're HIV, stigma that you're not straight. The pressure is just too much to carry. It's just too much to handle” (LBQ).

Stigmatizing community and social norms (meso level) construct sexual minorities as “demonic” and HIV as a “gay disease.” A participant articulated: “they see you being queer as being demonic or evil” (LBQ). This suggests felt-normative stigma shapes socioreligious attitudes towards sexual minorities. An IDU participant noted: “People made it [HIV] such a dirty thing because ‘it's a gay disease’.” This narrative reflects the symbolic stigma surrounding HIV as a “gay disease.” The convergence of homophobia and HIV-related stigma often resulted in enacted stigma such as violence: “if you're gay, lesbian, bisexual, or queer, and you're HIV positive, and you come out, you will be killed because it's like you're the one who start that virus” (LBQ). Several participants noted sexual violence motivated by homophobia or transphobia as a route of HIV infection.

Participants highlighted discrimination and exclusion within health care and HIV services (macro level). For example, a transgender participant's account of accessing health care services is indicative of enacted stigma: “I had a massive heart attack, a quadruple bypass, and I was going to go to rehab for two weeks. I had a hell of a time getting in there because their facilitator there said ‘we don't have any place to put something like that.’”

Exclusion and invisibility of LBQ and transgender women within HIV services signals heterosexism. Heterosexism refers to the normalization of heterosexuality and the subsequent invisibility of nonheterosexual sexualities [10],[55]. To illustrate, an LBQ participant explained: “the HIV prevention course only talks about condoms. So, you don't even know what else is dangerous for you as a queer woman, because all you know is heterosexual.” Services for HIV-positive women were described as predominately heterosexual, while LBQ and transgender women's services were primarily for non-PLHIV—rendering it difficult for participants to disclose both an HIV-positive serostatus and an LBQ/transgender identity in the same context.

### Sex Work Stigma: “They Treat You Like You're a Rat”

In the focus group conducted with women involved in sex work, participants highlighted felt-normative, enacted, and symbolic stigma experienced because of their HIV-positive serostatus and involvement in sex work. Participants' descriptions of micro-level stigma primarily involved interpersonal processes with family and friends. Several women explained that they lost friendships after disclosing an HIV positive serostatus and/or sex work involvement; this resulted in fear of disclosure. One participant described: “I've been positive for almost ten years. And I don't even tell half the people I know. As soon as I tell them they distance themselves.” This experience suggests felt-normative HIV-related stigma plays an integral role in shaping interpersonal relationships. As another woman explained: “with the drug abuse and the sex trade and being HIV [positive] they treat me totally different.” This account highlights the intersection of sex work stigma, HIV-related stigma, and drug use stigma. A participant articulated: “As someone who has worked in the sex trade, I keep it a secret a lot. Because even when I meet new friends, sometimes they're not the nicest people. And they'll say comments about working girls. They treat you like you're a rat. Like you're this small [makes a small space between her fingers]. Like you belong in a sewer.”

Fear of disclosing sex work involvement and HIV infection may also influence relationships with family and reduce access to care: “a lot of people don't even tell their families because of fear of being judged. So that can discourage people from disclosing and getting the proper supports and help that they need.” Sex work stigma and HIV-related stigma may also impact sex workers' children: “I do know from past experience with kids, and being a parent figure, that kids discriminated because their family members are in the sex trade. And even more, shun the kid out because the parent's got HIV. That is a big problem in public schools” (sex worker).

Participants highlighted community and social norms (meso level) that endorse symbolic HIV-related stigma directed toward sex workers. Symbolic stigma constructs sex workers as vectors of disease: “we all know that most people think that prostitutes are disease-carrying people. And if they really educated themselves around prostitution, they would know that prostitutes are pretty well-educated on protecting themselves on not getting diseases.” Sex workers may be incorrectly blamed as personally responsible for their HIV infection: “because you got it (HIV), you're careless. You're irresponsible. You're seen that way. But you're not.” Another woman articulated that people have the misconception that “You weren't protecting yourself” and clarified that “it could have been a broken condom from a long-term boyfriend. And all of a sudden everyone treats you like dirt.” Participants highlighted that sex workers are at risk for experiencing sexual violence: “there's a lot of date rapes.” Symbolic stigma may also exacerbate the intersection of sex work stigma, transphobia, and HIV-related stigma: “As soon as you say you're transgender, they automatically think you're a sex trade worker. And if you are a transgender sex trade worker, they automatically put you in a category where you're HIV [positive], and that's wrong” (transgender).

Participants discussed enacted sex work stigma within legal and health care systems (macro level). A transgender participant explained that she applied for refugee status but: “the immigration gave me a deportation order because they said I was a prostitute with HIV.” This suggests that legal protection for transgender human rights may be compromised because of sex work stigma and HIV-related stigma. Problems with medical systems were also articulated. One participant described: “I've been to a psychiatrist once and I hated it. I went there and I told him how I was feeling. I was very upset. I told him about working in the sex trade. And as soon as he heard that, he wanted to know, ‘How much were you charging? What kind of things were you doing? Describe it to me.’ I'm like, ‘Excuse me? Are you getting off on my stories? Are you serious? I'm not here to entertain you, I get paid to entertain. What the hell! I'm here because I need you to help me.’”

Participants reported being labeled and treated differently when receiving primary health care. As one woman recounted: “I had a friend who told one doctor that she worked in the sex trade and all of a sudden it's on her record. So every doctor she goes to now sees it on the paper. ‘Oh really, she's in the sex trade, let's test her for this and this and this.’ And she's like, ‘well I've already been tested for that. I don't have any of that stuff.’ But they'll do it every single time. They don't treat you like a normal patient.”

Another participant described being harassed by her family physician after disclosing her involvement in sex work: “I told one doctor at a clinic I was a dancer and I had pulled a muscle. After that he asked me out for a date, he asked me for my number. He called me at my house when I was 18 years old.” This narrative reflects the intersection of sex work stigma with gender discrimination. Such experiences led a sex worker to ask: “who do you get help from when you need help?”

### Coping Strategies Enacted by Women Living with HIV

Participants described varied coping strategies that helped them to manage the stress of living with intersectional stigma and to live positively. These strategies constitute resilience (micro), social networks (meso), and challenging stigma (macro). Table 4 highlights the focus groups that discussed each coping strategy (Table 4).

**Table 4:** Overview of focus group participants' (*n* = 104) descriptions of coping strategies.

Theme	Focus Groups Reporting Theme	Illustrative Quotations
Resilience	1, 2, 3, 8, 11, 13, 15	*“I always live with hope”* (African Caribbean participant, Ottawa)
Social Networks and Support Groups	5, 6, 7, 8, 9, 11, 13, 15	*“The person living with HIV wants to attend a support group when you need one, especially when you feel down”* (Latina participant)
Challenging Stigma	1, 2, 3, 4, 5, 7, 8, 11, 12, 13, 14	*“Stick together to fight the stigmas surrounding HIV individuals and fight for change”* (Transgender participant)

Focus group key: 1, African Caribbean (Ottawa); 2, African Caribbean (Toronto); 3, Asian and South Asian; 4, formerly incarcerated; 5, Francophone; 6, injection drug use; 7, Latina; 8, LBQ; 9, Northern Ontario medium-sized city; 10, Northern Ontario small-sized city; 11, sex work; 12, South Western Ontario medium-sized city; 13, transgender; 14, urban; 15, young women.

### Resilience: “I Always Live with Hope”

Resilient coping includes personality and attitudinal traits such as tenacity, optimism, and problem solving in difficult situations, and is associated with positive psychological outcomes [56],[57]. Participant testimonies reflected such traits. For example, a South Asian participant characterized coping as: “just being strong. You just have to think positive.” This account suggests optimism may be an important coping strategy. A sex worker's narrative reflects fighting back against internalized stigma and blame for childhood sexual abuse: “A lot of times people blame themselves for being abused. I was one of the very few people that didn't. I didn't think I was dirty. I just thought that person was a total piece of crap. And I felt that that had nothing to do with who I am.”

An LBQ participant highlighted the importance of: “self-determination, it's knowing what you really want in life and be focused on it.” An African Caribbean woman's testimony demonstrates tenacity and problem solving: “We have a lot to face. And, at the end of the day we sit and we feel depressed, we feel bad, but we have children to look after, send them off to school, some of us work. So, we get up and we face the day like nothing is going on, although we're hurting inside. I think that's our greatest strength.”

Faith and prayer may be personal resources integral to coping. A young woman articulated: “for me, praying is number one. I always keep praying to God that he would just guide me through all of this. Giving God praise for everything that he's done that's taken me through this whole thing.” This importance of spirituality was reiterated in a sex worker's account: “we went on a spiritual retreat and I feel that it would benefit a lot of people who have HIV because God's a strong presence.”

### Social Networks: “I Want to See More Peer Support”

Social networks of HIV-positive women emerged as a vital coping resource. A participant noted: “Sometimes the things that you have to face, you become so suicidal at many points in your life. You want to just end it. But then you meet friends; there are groups that can really help you to pull through. They give you strength” (LBQ). This narrative elucidates the benefits of support groups in helping HIV-positive women cope with intrapersonal stress. Support groups were described a way to combat feelings of isolation: “It's good [to] know you're not alone. And, I think people with HIV, the first thing within the first week, is to get into a group and talk” (Northern Ontario, small city). In addition to combating loneliness, support groups can provide a forum for sharing experiences and learning: “you can share experiences, so that you can help each other, so that you can feel better. Sometimes if you are alone you feel weak and you need somebody to say, ‘oh, I'm not alone in this world’” (young woman). Social support groups can be particularly important for sex workers who have multiple stigmatized identities: “because there is some similarity in lifestyle, people tend to feel more comfortable and open up when they're talking with somebody that's been through the same experiences” (sex worker). Because of these myriad benefits, more social support groups for HIV-positive women were called for: “I want to see more peer support” (IDU).

### Challenging Stigma: “Stand against the Stigmatization of Ourselves”

Challenging stigma was described as important for HIV-positive women's coping and empowerment; this involved engagement of HIV-positive women in fighting for equal rights. A Francophone African woman explained: “I think it's really important to stand against the stigmatization of ourselves and discrimination. You have the rights of a person just like everyone else.” Participants articulated “I think what needs to happen is political action” (urban) and advocacy “a woman with HIV advocating for somebody else who has HIV, that's a good idea” (formerly incarcerated). These accounts reflect the need for political mobilization. A critical component of challenging stigma may be education: “I think it is important to have education for the gay men or the lesbians to have a voice to fight stigma. No discrimination, no stigma” (LBQ). A sex worker described that community-based education can also address sexism and sex work stigma: “I think that if there is more education, the discrimination will also go away a little bit because people will have a better understanding of what these people actually go through. And that would be a big motivation if we went to schools about HIV, or specifically for sexual assault or sex trade work. And then everyone would not judge it and think of that person being dirty or that person is gross.”

Other participant narratives highlight the need for empowerment of HIV-positive women to challenge stigma in HIV services and research. Participants explained that HIV services: “should refocus on empowering women” (African Caribbean) and “we're the ones who are supposed to make the decisions” (Asian). This empowerment of HIV-positive women also applies to HIV research: “women should have more say in what research is being done” (sex worker). A sex worker explained: “I think we have to be more vocal. Otherwise they won't know what we go through and how we feel and what we need. And it would be nice to inform everyone of what's going on.” This statement reflects both the importance of articulating one's needs as well as educating others.

Reconfiguring research involves challenging the hierarchy in which HIV-positive women are research participants: “I really think that we have to be recognized for more than just as participants—we should be directing it” (industrial city). Involving women as directors of research and service providers also recognizes knowledge of lived experience: “how better can you help a woman with HIV, than have a woman counselor that has it and showing that they're functioning, they're healthy, and that they can survive in this world with HIV?” (IDU). It may also reduce social distance between service providers and participants: “I think that we could bring something to the table because we have firsthand knowledge and we've walked in those shoes. Working here [sex work agency] and being involved in prostitution myself—I'm able to connect with the other women” (sex worker).

## Discussion

Women living with HIV described interdependent and mutually constitutive relationships between social identities and structural inequities such as HIV-related stigma, racism, sexism and gender discrimination, homophobia, and transphobia. Our findings suggest that multiple forms of stigma, such as symbolic, internalized, and enacted, are associated with marginalized identities: HIV-positive serostatus, female gender, sex worker, sexual minority, transgender, and ethnic minorities. These forms of stigma operate across micro, meso, and macro levels—as do the coping strategies implemented by HIV-positive women. Marginalization within health care appears to be exacerbated for sexual minority and transgender women, sex workers, and women of color living with HIV. Most participants reported a lack of services tailored to meet the needs of women at mainstream HIV services and ASO. These overlapping, multilevel forms of stigma are representative of an intersectional [38],[45],[46] stigma model (see Figure 1).

On a micro, intrapersonal level, participants discussed how experiences of internalized and enacted stigma reduced self-esteem, self-worth, and for some participants increased depression or suicidality. These findings are supported by a large body of literature that suggests HIV-related stigma, racism, sexism, and homophobia are associated with deleterious mental health outcomes [18],[23]–[27],[32]. Resilience, optimism, and spirituality facilitated coping. These findings corroborate previous research that has highlighted the potential for adaptive coping styles to help people cope with stigma and discrimination [56]. On a meso level, participants reported social exclusion and ostracism on the basis of having one or more stigmatized identity. Conversely, joining social support groups emerged as an important coping resource for HIV-positive women. These findings are congruent with several studies that have found social support is associated with lower levels of HIV-related stigma [58],[59],[60] and homophobia [61]. On a macro level, HIV-positive women experienced discrimination and reduced access to care in social services, ASO, and health care. Participants' descriptions of institutionalized racism in health care corroborate research with African Caribbean women in Canada that highlighted discriminatory and inequitable treatment [3]–[6].

There were many limitations of this study, including a small sample size, nonrandom sampling, and different focus group sizes. Another limitation was the inability to differentiate responses on the basis of participants' multiple identities; as sociodemographic information was reported separately the participant's ethnicity in certain focus groups (e.g., LBQ, sex worker, IDU) was not accounted for when analyzing responses. Therefore, unless another identity was explicitly referred to, it was not possible to differentiate experiences on the basis of multiple identities beyond the focus group population. These limitations limit the generalizability of findings to other samples of HIV-positive women. The congruency between study findings and previous research with HIV-positive women, however, suggests important similarities in experiences of stigma and coping.

A strength of this study is its inclusion of diverse HIV-positive women including ethno-racial minorities, rural and urban women, and LBQ, IDU, and sex workers. There were striking similarities between participants across the 15 focus groups: all groups highlighted HIV-related stigma; most discussed sexism and gender discrimination; and the majority of groups endorsed social support groups and advocacy as coping resources. Homophobia and transphobia were discussed by the LBQ, transgender, IDU, and sex worker focus groups; these themes may be more pronounced among populations targeted by symbolic stigma that associates HIV with “deviant” sexuality and behavior.

### Conclusion

Previous conceptualizations of HIV-related stigma suggest that racism, sexism, and preexisting stigma towards sex workers and men who have sex with men are predisposing facilitators of HIV-related stigma [7],[10],[21],[34]. The current study findings expand upon these frameworks by: (1) suggesting that women also experience predisposing stigma on the basis of sexual orientation, female gender, and transgender identity; (2) illuminating the micro, meso, and macro levels of stigma; and (3) focusing on multilevel coping strategies to challenge stigma. This study's conceptualization of “intersectional stigma” enriches the discussion of the complexity of stigma and myriad coping strategies.

A clear understanding of the complexity of stigma can inform the development, implementation, and evaluation of stigma reduction interventions and health promotion initiatives tailored to meet the needs of HIV-positive women [7],[11],[54],[62]. Researchers, practitioners, and policy makers should consider comprehensive interventions that are multilevel and address intersectional forms of stigma. Individual and community interventions to challenge stigma may include counseling, education, information, and mass media campaigns [7],[63], and programs to increase the quality of care for PLHIV from family and friends [8],[63]. Structural interventions to challenge HIV-related stigma, racism, sexism, and homophobia could include antidiscrimination training using an intersectional approach for health care providers [7],[17],[64]–[68].

The notion of “cultural discordance”—cultural differences and misunderstandings between patients and health care providers—has been highlighted in previous research with African Americans accessing diabetes care [16]. In the present study disconnections between service providers and HIV-positive women of color, sex workers, transgender, and sexual minority women suggest the need for culture, race/ethnicity, sexual orientation, and gender to be better integrated into health care and HIV support services. It is not only imperative to address stigma and discrimination, but also the discordance between the services offered and the needs of diverse HIV-positive women.

Overall, this investigation highlights a complex system of intersectional stigma that necessitates multifaceted strategies to promote health equity for HIV-positive women. Findings can inform treatment, care, and support guidelines and practice recommendations for health care practitioners, social workers, HIV prevention and support workers, and mental health specialists working with HIV-positive women. Designing programs that attend to gender and gender identity, race/ethnicity, sex worker needs, and sexual orientation may enhance access to, and uptake of, services. Strategies that support HIV-positive women by fostering resilience and social support, while simultaneously challenging stigmatizing community/social norms and structural inequities, can facilitate health promotion and social justice.

## Abbreviations

ASO: AIDS service organization
CAB: community advisory board
IDU: injection drug using
LBQ: lesbian, bisexual, and queer
PLHIV: people living with HIV
PRA: peer research assistant
